# Cost and effectiveness of microwave ablation versus video-assisted thoracoscopic surgical resection for ground-glass nodule lung adenocarcinoma

**DOI:** 10.3389/fonc.2022.962630

**Published:** 2022-10-05

**Authors:** Xiaoying Han, Zhigang Wei, Zhenxing Zhao, Xia Yang, Xin Ye

**Affiliations:** ^1^ Department of Oncology, Shandong Provincial Hospital Affiliated to Shandong First Medical University, Jinan, China; ^2^ Department of Oncology, The First Affiliated Hospital of Shandong First Medical University and Shandong Provincial Qianfoshan Hospital, Shandong Key Laboratory of Rheumatic Disease and Translational Medicine, Shandong Lung Cancer Institute, Jinan, China; ^3^ Cheeloo College of Medicine, Shandong University, Jinan, China; ^4^ Shandong First Medical University, Jinan, China

**Keywords:** lung adenocarcinoma, microwave ablation, video-assisted thoracoscopic surgery, ground-glass nodule, cost effectiveness

## Abstract

**Purpose:**

To retrospectively evaluate the cost and effectiveness in consecutive patients with ground-glass nodules (GGNs) treated with video-assisted thoracoscopic surgery (VATS; i.e., wedge resection or segmentectomy) or microwave ablation (MWA).

**Materials and methods:**

From May 2017 to April 2019, 204 patients who met our study inclusion criteria were treated with VATS (n = 103) and MWA (n = 101). We calculated the rate of 3-year overall survival (OS), local progression-free survival (LPFS), and cancer−specific survival (CSS), as well as the cost during hospitalization and the length of hospital stay.

**Results:**

The rates of 3-year OS, LPFS, and CSS were 100%, 98.9%, and 100%, respectively, in the VATS group and 100%, 100% (p = 0.423), and 100%, respectively, in the MWA group. The median cost of VATS vs. MWA was RMB 54,314.36 vs. RMB 21,464.98 (p < 0.001). The length of hospital stay in the VATS vs. MWA group was 10.0 vs. 6.0 d (p < 0.001).

**Conclusions:**

MWA had similar rates of 3-year OS, LPFS, and CSS for patients with GGNs and a dramatically lower cost and shorter hospital stay compared with VATS. Based on efficacy and cost, MWA provides an alternative treatment option for patients with GGNs.

## Introduction

Although the incidence of lung cancer is the second-highest of all cancers globally (1), it is the leading cause of cancer deaths in both China and the USA. By 2022, China and the USA are expected to have approximately 870,982 and 238,032 new lung cancer cases and 766,898 and 144,913 lung cancer deaths, respectively (2). Early detection, early diagnosis, and early treatment are critical for mortality reduction. In 2011, the National Lung Screening Trial was the first to report that lung cancer mortality in high-risk populations could be reduced by 20% by screening with low-dose computed tomography (LDCT) instead of standard chest X-ray (3). The widespread application of LDCT has increased the detection rate of asymptomatic pulmonary nodules, and it is estimated that pulmonary nodules are detected in 20%–80% of patients screened in China (4–7). A pulmonary nodule (ground-glass nodule, GGN) is often considered a predictor of a precancerous lesion or early-stage lung cancer. However, more than 97% of GGNs identified by LDCT are benign, with only 0.7%–2.3% of GGNs diagnosed as lung cancer (8–10). GGN lung adenocarcinoma is characterized by indolent development with few distant metastases and has a favorable prognosis, with a 5-year survival rate of 100% after surgery (11–16). Thus, GGN lung adenocarcinoma is deemed a special subtype of lung cancer that differs from traditional early-stage lung cancer.

The primary therapy for cases of GGN lung adenocarcinoma is surgical resection (video-assisted thoracoscopic surgery, VATS) with curative intent. However, there are several limitations to the application of VATS (10, 17). First, premature surgical intervention for GGNs, particularly precancerous lesions, leads to early and unnecessary organ damage and loss of lung function. Moreover, early surgery does significantly improve the overall survival (OS) of patients when compared with those who choose follow-up and elective surgery as interventions. Second, there are no clear selection criteria for surgical intervention of multiple pulmonary nodules and no principles for the follow-up management of residual nodules. Third, because the preoperative diagnosis of pulmonary nodules is based on imaging results and not pathological evidence, surgical resection of pulmonary nodules may be unnecessary and cause needless postoperative complications if the lesions are benign. Fourth, as the population ages, an increasing number of patients aged >75 years are being diagnosed with early-stage lung cancer, and surgery is almost impossible in these cases. Therefore, many novel local treatment approaches have been developed, including image-guided thermal ablation therapy. This precise and minimally invasive technique has been used to treat early-stage lung cancer and includes radiofrequency ablation, microwave ablation (MWA), cryoablation, and laser ablation. MWA was first applied to lung tumors in 2002 (18), and its use has increased over the years (19).

MWA has been proved one of the effective methods to treat GGN lung adenocarcinoma (20–24). Although cost-effectiveness analysis is a proven analytic technique to assess the relative benefit of a given treatment strategy, the cost-effectiveness analysis of MWA in GGN lung adenocarcinoma patients has remained unexplored. Therefore, we performed a cost-effectiveness analysis comparing VATS and MWA for patients with GGN lung adenocarcinoma and analyzed 3-year OS, local progression-free survival (LPFS), and cancer−specific survival (CSS), as well as the length of hospital stay and cost during hospitalization.

## Materials and methods

### Patient inclusion and exclusion criteria

This retrospective study was approved by the Ethics Committee of Shandong Provincial Hospital, affiliated with Shandong First Medical University. The study complied with the ethical principles of the World Medical Association’s Declaration of Helsinki. Written informed consent was obtained from all participants.

From May 2017 to April 2019, 204 consecutive patients with GGNs confirmed by contrast-enhanced computed tomography (CT) and pathology were treated in our institute. Of them, 103 underwent VATS (VATS group) and 101 underwent CT-guided MWA (MWA group). The treatment decisions for each patient were made by a multidisciplinary tumor board that included medical oncologists, thoracic surgeons, respiratory physicians, radiologists, and pathologists who reviewed the medical history, physical examination results, and recent imaging studies. For those patients undergoing MWA, contrast-enhanced chest CT (within 2 weeks before MWA) was considered a key imaging assessment in revealing the tumor size, location, and the relationship with neighboring vital organs, blood vessels, the trachea, or bronchi.

The study inclusion criteria were as follows: (1) Eastern Cooperative Oncology Group performance status 0–2; (2) patients aged ≥18 years and nonpregnant females; (3) a solitary pure GGN or mixed ground-glass opacities (mGGOs; lesions with a ratio of consolidation diameter to tumor diameter of <0.25 at a slice thickness ≤ 1 mm) demonstrated by CT, with a diameter ≤30 mm and without lymph node involvement or distant metastasis; (4) histological diagnosis of adenocarcinoma in situ, minimally invasive adenocarcinoma or invasive adenocarcinoma (25) through percutaneous coaxial needle biopsy (26–28) or using a postoperative specimen; (5) an expected lifespan of ≥12 months; and (6) no chemotherapy or radiotherapy performed after the procedure. The exclusion criteria were as follows: (1) the presence of regional lymph node metastasis or distant metastasis verified by enhanced CT, positron emission tomography-CT, and enhanced magnetic resonance imaging; (2) GGNs accompanied by other malignant tumors; and (3) untreatable coagulopathies and/or platelet count < 50 × 10^9^/L. The histological diagnoses were either confirmed by conventional paraffin sections in separate procedures or by frozen sections and postprocedural paraffin sections in the same procedure.

### Instrument and MWA procedure

CT (GE Lightspeed 64 VCT, General Electric, or NeuViz 64, Neusoft Medical Systems) was used to guide MWA, which was performed with an MTC-3C MWA system (Vison-China Medical Devices R&D Center), ECO-100A1 MWA system (ECO Medical Instrument Co., Ltd.), or KY-2450B MWA system (CANYOU Medical Inc.) at a frequency of 2450 ± 50 MHz. The adjustable continuous wave output power ranged from 0 to 100 W. For the microwave antenna, the effective length was 100–180 mm, and the outside diameter was 14–19 G (19 G antenna has the advantages of high puncture accuracy and few complications), with a 1.5 cm radiating tip (tapered end). The surface temperature of the antennae was cooled with a water circulation cooling system.

**Figure 1:**
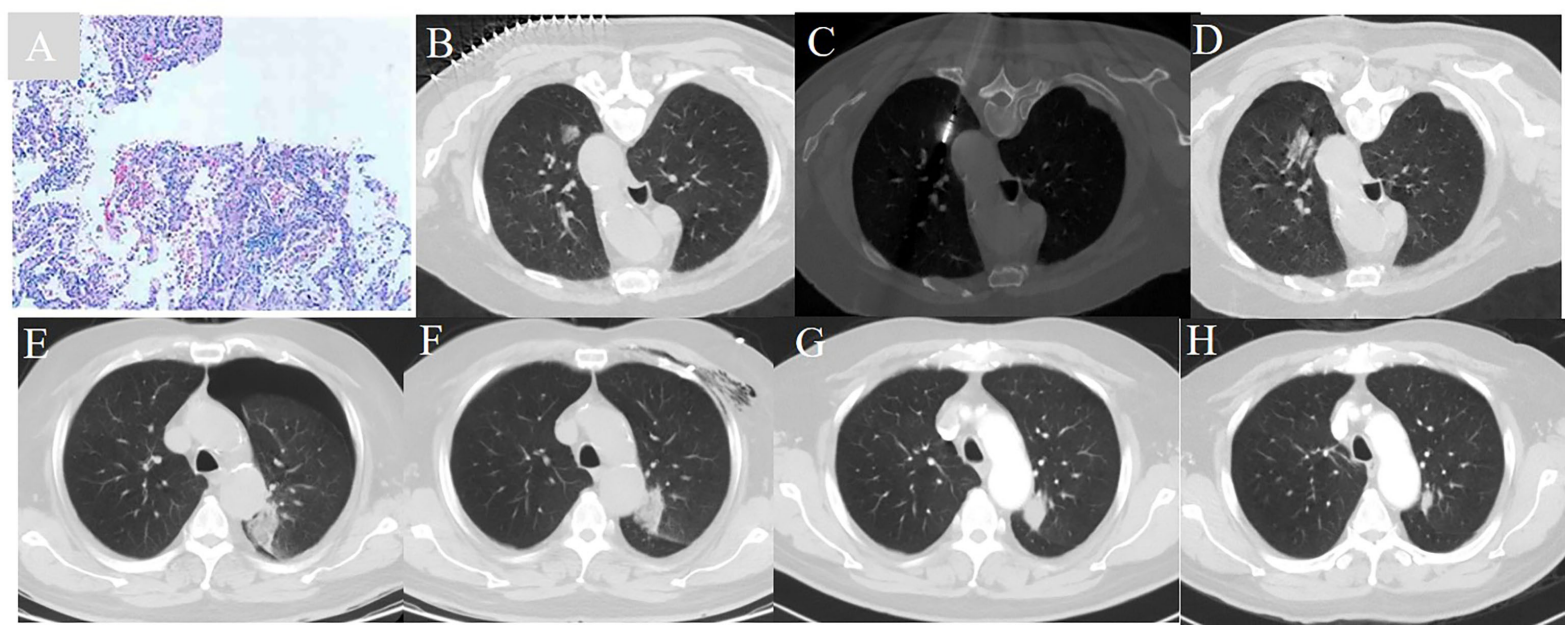
A 72 year-old man with a history of COPD and a FEV1 value of 51.2% underwent CT guided microwave ablation after pathological diagnosis of adenocarcinoma in situ. **(A)** Pathology verified adenocarcinoma in situ. **(B)** A ground glass nodule with a diameter of 1.7 cm located in the left upper lobe. **(C)** MWA was conducted with the power of 65 W for a total of 4.5 min. **(D)** The immediate CT scan showed the nodule was surpassed by the exudative change. **(E)** A CT scan showed moderate pneumothorax 24 hours post-ablation. **(F)** Pneumothorax was resolved 72 hours post-ablation. **(G)** The ablative zone shrank and increased in density one month post-ablation. **(H)** The CT ablative zone further reduced one year post-ablation.

We performed our standard MWA procedure as per our previous descriptions (29–31). A treatment plan was designed immediately following pre-procedural CT and was based on the tumor location and size, as well as adjacent structures. The appropriate body placement, puncture site on the body surface, optimal puncture trajectory, and antenna number were confirmed. All percutaneous MWA procedures were performed using a sterile technique under local anesthesia, with the patients under moderate sedation. After achieving satisfactory anesthesia, the procedure was performed by positioning the antenna into the initially planned site. After using CT to ascertain that the antennae were properly positioned, we performed MWA at the predetermined power and time. The range of the ablation zone was monitored in real-time on CT, and when it was 5–10 mm beyond the lesion boundary, the ablation procedure was terminated, the antennae were immediately withdrawn from the lesion, and the puncture wound was disinfected and bandaged. At the end of the procedure, a repeat whole-lung CT scan was performed to assess technical success and immediate complications. The procedure was defined as a technical success when the tumor was treated according to protocol and completely covered (i.e., the ablation zone completely overlapped or encompassed the target tumor plus an ablative margin). The patient’s electrocardiographic tracing, heart rate, respiratory rate, oxygen saturation, and blood pressure were continuously monitored throughout the MWA session and for an additional 6 h after their safe return to the ward (**Figure 1**).

### Equipment and VATS procedure

The VATS procedure was performed using an IMAGE1 HD video system (Karl Storz, Inc., Germany), a Harmonic ultrasonic scalpel (Ethicon Endo-Surgery, LLC, Puerto Rico, USA), and an Endo GIA Ultra Universal Stapler (Covetingly, MA, USA).

**Figure 2:**
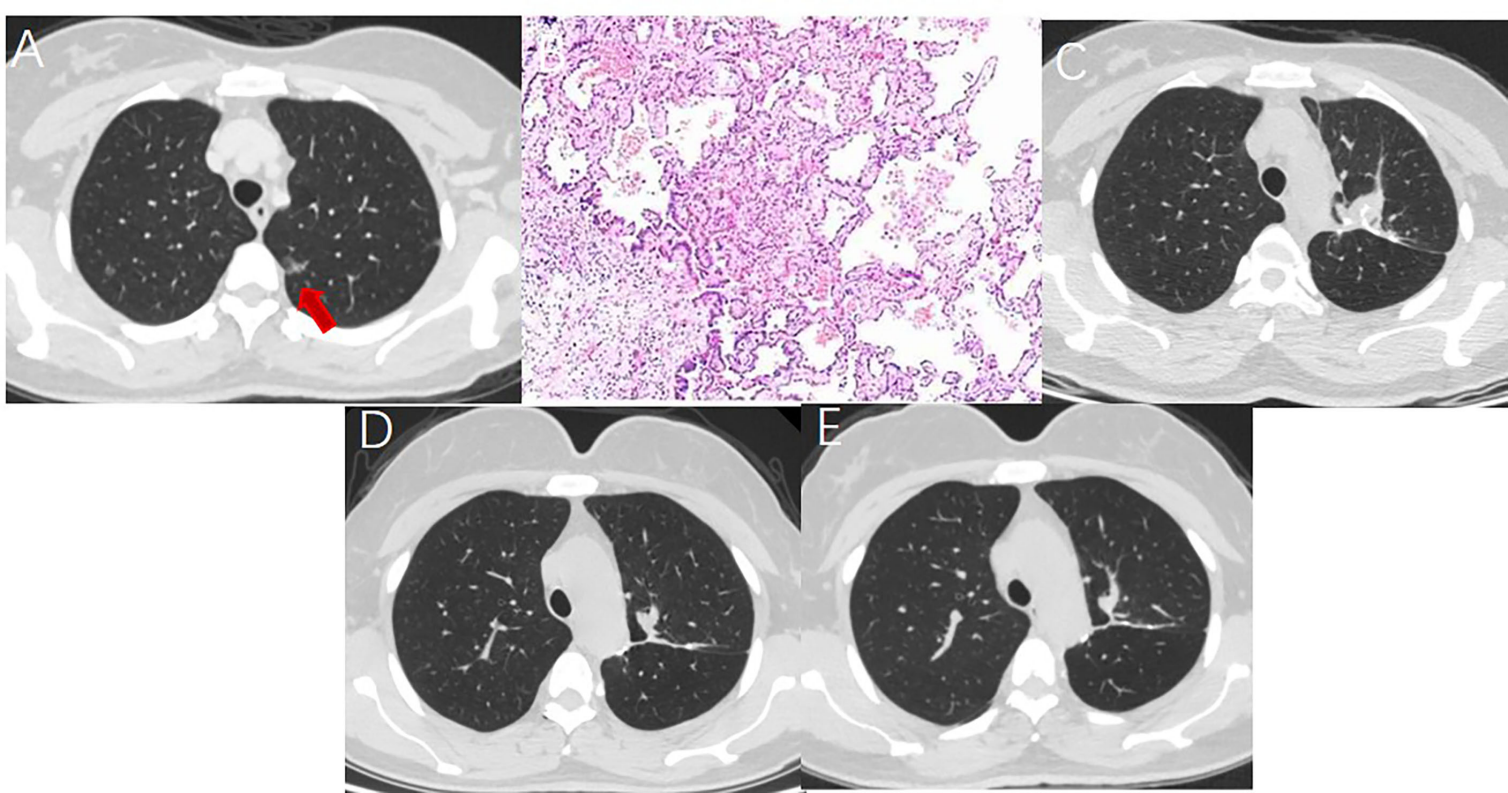
A 52 year-old woman with multiple GGNs underwent resection of apical posterior segment of left upper lobe and lymph node sampling. **(A)** A ground glass nodule with a diameter of 1.5cm located in the left upper lobe. **(B)** Postoperative pathology showed minimal invasive adenocarcinoma with no lymph node metastasis. **(C)** Soft tissue-density mass and linear stapler can be seen in the operation area on the CT image one month after surgery. **(D)** The soft tissue density mass in the operation area shrank 7 months after surgery. **(E)** The operation area formed fibrous scar 14 months after surgery.

The VATS procedure was performed under general anesthesia with single-lung ventilation, which was accomplished with either dual-lumen endotracheal tubes or single-lumen tubes and bronchial blockers. The patient was placed in a left-sided lateral position with the right hemithorax slightly overextended so that the intercostal spaces could be expanded to facilitate the operation. A single incision approximately 4 cm long was made along the fifth intercostal space just anterior to the midaxillary line. Hilar dissection was performed through the anterior incision. Dissection of the pulmonary vessels and bronchi was performed in the same manner as in open surgery. Endoscopic linear staplers were used for individual vessel and bronchial ligation. Parenchymal resection margin ≥ 2 cm should be achieved. The lobe was placed in a specimen bag for retrieval after complete resection. Mediastinal lymph nodes were not dissected or only sampled. After the operation, a 28 F chest tube was placed at the apex of the thorax, and an 18 F soft tube was left in the part of the thorax most dependent on drainage. No extra incision was made for drainage. Both chest tubes were connected to a water seal drainage system without suction. The 28 F tube was removed 48 h after surgery when there was no air leak and the lung showed good expansion. The 18 F tube was kept until discharge, when the drainage was <150 mL/d (32–34) (**Figure 2**).

### Follow-up and outcome assessment

Contrast-enhanced chest CT was performed monthly for the first 3 months post-MWA and -VATS and then every 3 months for the first year. Thereafter, the follow-up intervals were extended to 6 months. For those patients who underwent MWA, we assessed the local ablation effect by signs and dynamic changes in the lesion on a series of repeated contrast-enhanced CT scans and used the lesion at 4–6 weeks post-MWA as the baseline for comparisons. The local effect of the ablative response included complete ablation, incomplete ablation, and local progression (35, 36). For the patients who underwent VATS, we applied the Response Evaluation Criteria in Solid Tumors version 1.1 to assess responses (37). We used the follow−up results to assess the 3-year LPFS, CSS, and OS. LPFS was defined as the time interval from the initial MWA or VATS to the first radiologic evidence of local progression. CSS was defined as the time interval from the initial MWA or VATS to 3 years or cancer−related death. The OS was defined as the time interval from the initial MWA or VATS to death from any cause. We also recorded the length of the hospital stay. The during of hospital stay including both the periods before and after ablation

### Cost

We identified the direct and indirect costs of MWA and VATS but only included the direct costs during hospitalization in this study. The direct costs included the cost of MWA or VATS treatment during hospitalization, mainly comprising the fees for medicine, laboratory tests, examination, anesthesia, ICU, operation, medical supplies, blood transfusion, etc. (38–41). The cost of hospitalization was determined by reviewing the billing details of hospitalization expenses.

### Complication assessment

Complications were assessed based on the number of ablation procedures. The severity of injuries to patients was classified as major or minor according to the Cirse Quality Assurance Document and Standards for Classification of Complications: The Cirse Classification System (42, 43). Major complications were defined as events that led to substantial morbidity and disability (e.g., unexpected loss of an organ), an increased level of care, hospital admission, or a substantially prolonged hospital stay (classifications 3–5). This also included any case where a blood transfusion or an interventional drainage procedure were required. Any patient’s death within 30 d after image-guided tumor ablation was addressed (classification 6). All other events were considered minor complications (classifications 1–2). VATS complications were classified according to surgical complications (44).

### Statistical analysis

All statistical analyses were performed using SPSS 17.0 software (SPSS, Chicago, IL, USA). Categorical variables were presented as numbers and percentages. Continuous data were presented as means and standard deviations. We compared local and control rates using the chi-square test and length of hospital stay and cost using an independent *t*-test. Survival curves were constructed using the Kaplan–Meier method and compared by the log-rank test. A p value < 0.05 was considered statistically significant.

## Results

As of April 1, 2022, no patients were lost follow−up, and the median follow-up time was 40.1 and 42.6 months in the VATS and MWA groups, respectively. All patients are under clinical observation and have not received any antitumor treatments, such as stereotactic radiation therapy, chemotherapy, targeted therapy, and immunotherapy.

Compared with the VATS group, those in the MWA group were older (66.40 vs. 56.70 years), predominantly male (54.5% vs. 40.8%), and had higher comorbidity of cardiovascular (28.7% vs. 9.7%) and pulmonary disease (33.7% vs. 12.6%). Other characteristics were comparable between the two groups (**Table 1**).

**Table 1:** Baseline characteristics of enrolled patients.

Characteristics	MWA group (%) N = 101	VATS group (%) N = 103	*p*
Age (years, range)	66.40 ± 11.35 (27,88)	56.70 ± 9.01 (31,82)	<0.001
Sex			0.050
Male	55 (54.5)	42 (40.8)	
Female	46 (45.5)	61 (59.2)	
ECOG			NA
0–1	101 (100.0)	103 (100.0)	
2	0 (0.0)	0 (0.0)	
Comorbidity			
Cardiovascular diseases	29 (28.7)	10 (9.7)	0.001
Pulmonary diseases	34 (33.7)	14 (13.6)	0.001
Diabetes	16 (15.8)	13 (12.6)	0.510
Smoking			0.148
No	64 (63.4)	75 (72.8)	
Yes	37 (36.6)	28 (27.2)	
Location of GGN			0.068
Right upper lobe	32 (31.7)	32 (31.1)	
Right middle lobe	4 (4.0)	0 (0.0)	
Right lower lobe	18 (17.8)	25 (24.3)	
Left upper lobe	27 (26.7)	35 (34.0)	
Left lower lobe	20 (19.8)	11 (10.7)	
Size of GGN (mm)			<0.001
Mean ± SD (range)	16 (15.8)	28 (27.2)	
Size of GGN (mm)			NA
≤ 10	16 (15.8)	28 (27.2)	
> 10, ≤ 20	51 (50.5)	65 (63.1)	
> 20	34 (33.7)	10 (9.7)	
CT finding (GGN type)			0.061
pGGN	35 (34.7)	49 (47.6)	
mGGN	66 (65.3)	54 (52.4)	
Histology of GGN			<0.001
AIS	28 (27.7)	32 (31.1)	
MIA	13 (12.9)	44 (42.7)	
IA	60 (59.4)	27 (26.2)	
T stage at diagnosis			0.069
T1a	8 (13.3)	2 (7.4)	
T1b	31 (51.7)	21 (77.8)	
T1c	21 (35.0)	4 (14.8)	

AAH, atypical adenomatous hyperplasia; AIS, adenocarcinoma in situ; CT, computed tomography; GGN, ground glass nodule; IA, invasive adenocarcinomas; mGGO, mixed ground glass opacity; MIA, minimally invasive adenocarcinomas; MWA, microwave ablation; pGGN, pure ground glass nodule; SD, standard deviation; VATS, Video-Assisted Thoracoscopic Surgery. NA, not application.

### Survival

In the VATS group, 1 (1.0%) patient showed local tumor progression 36.4 months after VATS and was subsequently treated with radiation therapy. At the 3−year follow−up, 103 of 104 VATS tumors were controlled. The 3−year LPFS, CSS, and OS rates were 98.9%, 100%, and 100%, respectively. No mediastinal lymph node and distant metastases were observed in any patients in the VATS or MWA groups.

In the MWA group, 4 (4.0%) patients showed local tumor progression. These patients underwent a second MWA, and complete ablation was achieved. At the 3−year follow−up, all ablated tumors (105 sessions) were under control. The 3−year LPFS, CSS, and OS rates were all 100%. There was no significant difference in 3-year LPFS (p = 0.423), OS(p=1.000), and CSS(p=1.000) between the VATS and MWA groups.

### Cost and length of hospital stay

The mean length of hospital stay for the MWA group was significantly lower compared with the VATS group 6.0 vs. 10.0 d(p<0.001). Furthermore, the median cost, medicine fee, and medical supplies fee for the MWA group was lower than the VATS group (RMB 21,464.98 vs. RMB 54,314.36, RMB 2,516.23 vs. RMB 8,970.04, and RMB 7,568.14 vs. RMB 27,167.25, respectively; p < 0.001 for all). However, the laboratory and examination fees were similar between the two groups (5,252.70 vs. 6,103.50, p = 0.191). Other fees, including the operation, anesthesia, ICU, and blood transfusion fees, were only observed in the VATS group (**Table 2**).

**Table 2:** The comparation of cost between the two groups.

	MWA group	VATS group	P
Hospital stay	6.00 (5.00,9.00)	11.00 (8.00,12.00)	<0.001
Medicine fee	2516.23 (1588.50,4293.94)	8970.04 (6776.80,11659.47)	<0.001
Laboratory and examination fee	5252.70 (4647.95,11499.00)	6103.50 (5095.40,7576.40)	0.191
Operation fee	–	5455.00 (4690.00,5455.00)	<0.001
Anesthesia fee	–	3383.05 (3200.05,3545.08)	–
ICU fee	–	0.00 (0.00,0.00)	–
Medical supplies fee	7568.14 (6571.54,11469.71)	27167.25 (21147.66,31708.96)	<0.001
Blood transfusion fee	–	790.00 (150.00,950.00)	–
All	21464.98 (17373.77, 26576.84)	54314.36 (47673.58,62733.88)	<0.001

ICU, intense care unit; MWA, microwave ablation; VATS, Video-Assisted Thoracoscopic Surgery.

### Complications

The MWA and VATS procedures were successfully performed in all patients. Perioperative complications are listed in **Table 3**. Infection occurred in two patients in the VATS group. No respiratory failure was observed in either group. There were 51 (50.5%) and 7 (6.8%) patients in the MWA and VATS groups, respectively, who suffered slight pneumothorax after the procedure (p < 0.001). Only 10 patients (19.6) underwent classification 3 pneumothorax and chest tube drainage was conducted in the MWA group. No one underwent respiratory failure was observed in both groups.

**Table 3:** The complications of enrolled patients.

Characteristic	MWA group	VATS group	p
Mortality	0 (0.0)	0 (0.0)	NA
Pain (post procedure)	3 (3.0)	5 (4.9)	0.721
Pneumothorax	51 (50.5)	7 (6.8)	<0.001
Pleural effusion	55 (54.5)	48 (46.6)	0.262
Infection	3 (3.0)	2 (1.9)	0.681
All	73 (72.3)	52 (50.0)	0.001

MWA, microwave ablation; NA, not application; VATS, Video-Assisted Thoracoscopic Surgery.

Forced expiratory volume in the first second and forced vital capacity was similar between pre-ablation and one-month post-ablation. There were no significant differences in the incidence of mortality, pleural effusion, coronary/cerebral vascular events, and bleeding requiring reoperation between the two groups.

## Discussion

During the past 2 decades, VATS has been established as the gold-standard surgical approach for lobectomy in patients with early-stage non-small-cell lung carcinoma (including GGN lung adenocarcinoma). VATS shows various advantages over open surgery, such as decreased blood loss, less pain, shorter hospital stay, more rapid recovery, preserved postoperative pulmonary function, and decreased inflammatory response. Furthermore, the early and late outcomes of VATS are comparable to or even superior to those of open thoracotomy (45–50). At present, many clinical studies have reported the efficacy and safety of percutaneous CT-guided MWA to treat GGN lesions (20–22, 51, 52). However, few studies have compared the differences in clinical outcomes, cost, and complications between MWA and VATS for GGN lung adenocarcinoma.

The follow−up results of this study showed a 3-year OS, LPFS, and CSS rate of 100% vs. 100%, 98.9% vs. 100%, and 100% vs. 100% in the VATS vs. MWA groups, respectively. No significant differences were observed in log-rank analysis between the groups (p = 0.171). Our findings suggest that MWA has similar efficacy to VATS in patients with GGN lung adenocarcinoma. Wang et al. reported a similar finding (52). For both MWA and VATS in the treatment of GGOs, the 3 year-OS and CSS were both 100%, local disease progression was only observed in 5 patients, which was significantly superior to those with solid tumors.

Our results demonstrate that MWA is less costly and results in a better quality of life (fewer complications) compared with VATS for patients with GGN lung adenocarcinoma (**Table 3**). The primary cost differences were associated with expenses related to the anesthesia, ICU, medical supplies, blood transfusion, and medicine fees. This suggests the superiority of the MWA approach compared with VATS and should consequently, from an economic standpoint, not discourage physicians or thoracic surgeons from implementing MWA in their practice. The length of hospital stay in the MWA group was shorter than in the VATS group because MWA for patients with GGN lung adenocarcinoma does not require general anesthesia or a stay in ICU. Additionally, there is less trauma and faster recovery. Furthermore, there are few serious complications post-MWA, which is one of the reasons for the shorter hospital stay.

For patients with multiple GGOs, ENB-guided microwave ablation combined with uniportal VATS is a treatment regimen. However, compared with CT guidance, the bronchoscopy guidance means the longer treatment interval and more cost. For those lesions located in the peripheral of the lung, CT guided MWA was superior to bronchoscopy guided MWA. However, for the GGOs located in the middle of the lung, the bronchoscopy guided MWA had the advantage (53). For patients with GGOs and contradiction to surgery, cryoablation was a treatment regimen. Compared with radical surgery, the cryoablation procedures are associated with less trauma, high efficacy rates, and fast recovery and is therefore applicable across a wide range of patient populations (54, 55).

Although compared with VATS, MWA had more pneumothorax, those with classification 3 pneumothorax was few. The pneumothorax did not affect the respiratory function and no respiratory failure was observed. So, the MWA in the treatment of GGN was safe.

There were some limitations to this study. First, it was a retrospective single-institution study. Second, there was a relatively small number of cases and a relatively short follow-up period. Third, we used the direct cost of medical treatment in a single hospital and did not calculate the indirect costs. Additionally, the cost of hospital readmission for serious complications related to MWA and VATS treatment after discharge and the cost of outpatient treatment during follow-up were not calculated. Fourth, due to the unbalanced development of MWA and VATS technology in different regions in China, the study population is not representative of the other regions of China. Therefore, a prospective, multicenter, randomized controlled study is necessary to evaluate cost and effectiveness in patients with early-stage GGN lung adenocarcinoma treated with MWA vs. VATS.

In conclusion, this study suggests MWA as an effective and safe option to treat early-stage GGN lung adenocarcinoma, with efficacy similar to VATS, a lower cost, and a shorter hospital stay. Despite being a newly incorporated technology, MWA provides an alternative treatment for this disease. In the future, a prospective, multicenter, randomized controlled study is necessary to evaluate the cost and efficacy of MWA in the treatment of early-stage GGN lung adenocarcinoma.

## Data availability statement

The original contributions presented in the study are included in the article/supplementary material. Further inquiries can be directed to the corresponding authors.

## Ethics statement

The studies involving human participants were reviewed and approved by Shandong Provincial Hospital affiliated to Shandong First Medical University. Written informed consent to participate in this study was provided by the participants’ legal guardian/next of kin.

## Author contributions

XYa and XYe: Conceptualization, data curation, methodology, and writing—original draft. XH, ZW, ZZ, XYa, and XYe: Data curation, methodology, resources, and writing—final draft. All authors contributed to the article and approved the submitted version.

## Funding

This study received funding from the National Natural Science Foundation of China (81502610 and 82072028) and the Shandong Provincial Natural Science Foundation, China (ZR2021MH143 and ZR2020MH294).

## Conflict of interest

The authors declare that the research was conducted in the absence of any commercial or financial relationships that could be construed as a potential conflict of interest.

## Publisher’s note

All claims expressed in this article are solely those of the authors and do not necessarily represent those of their affiliated organizations, or those of the publisher, the editors and the reviewers. Any product that may be evaluated in this article, or claim that may be made by its manufacturer, is not guaranteed or endorsed by the publisher.
